# Hidden diversity and host specificity of bat trypanosomes in East and Central Africa

**DOI:** 10.1007/s00436-025-08547-4

**Published:** 2025-09-11

**Authors:** Sadic Waswa Babyesiza, Alena Fornůsková, Abdul Katakweba, Eric Kigai, Kristýna Hadová, Jean Luther Ngbangui Kaguendo, Labo Dieu-béni Sosthène Bonaventure, Primael Tabiti, Thierry Aebischer, Joëlle Goüy de Bellocq, Josef Bryja, Jan Votýpka

**Affiliations:** 1 Institute of Vertebrate Biology of the Czech Academy of Sciences, Brno, Czech Republic; 2 Department of Zoology, Entomology and Fisheries Science, Makerere University, Kampala, Uganda; 3 Africa Centre of Excellence for Innovative Rodent Pest Management and Biosensor Technology Development (ACE IRPM&BTD), Institute of Pest Management Centre, Sokoine University of Agriculture, Morogoro, Tanzania; 4 University of Bangui, Bangui, Central African Republic; 5 African Parks, Johannesburg, South Africa; 6 Department of Parasitology, Faculty of Science, Charles University, Prague, Czech Republic; 7 Institute of Parasitology, Biology Centre, Czech Academy of Sciences, České Budějovice, Czech Republic

**Keywords:** Bat trypanosomes, Phylogenetic diversity, Host specificity, African Chiroptera

## Abstract

Bats are recognized as key reservoir hosts for a variety of trypanosomes, yet their full diversity and host associations remain underexplored in Africa. In this study, we investigated the diversity and phylogenetic relationships of trypanosomes infecting bats in Uganda and the Central African Republic (CAR) using molecular tools. A total of 22 distinct genotypes were identified, clustering into seven well-supported phylogroups, including known species such as *Trypanosoma dionisii*, *T. livingstonei*, *T. vespertilionis*, and *T. erneyi*, alongside several novel lineages. Notably, *T. erneyi* was restricted to *Mops condylurus*, reaffirming its narrow host specificity. Phylogenetic analyses revealed substantial intraspecific diversity in *T. livingstonei* and *T. vespertilionis*, suggesting potential cryptic species, while *T. dionisii* exhibited limited genetic divergence across a broad geographic range. Intriguingly, a unique and highly diverse phylogroup was detected exclusively in fruit bats (Pteropodidae) from Uganda, closely related to the Australian *T. teixeirae*, and likely represents a novel species. The absence of trypanosomes in fruit bats from CAR, despite similar sampling effort, hints at ecological or evolutionary factors shaping parasite distribution. Our findings expand the current knowledge of bat-trypanosome diversity and highlight the importance of African bat populations in understanding the evolutionary dynamics of trypanosomes.

**Supplementary Information:**

The online version contains supplementary material available at 10.1007/s00436-025-08547-4.

## Introduction

Bats (order Chiroptera) are among the most diverse and widely distributed groups of mammals globally (Burgin et al. 2018). With over 1400 species documented, bats account for nearly a quarter of all mammalian species and inhabit a wide range of ecosystems, from tropical rainforests to arid deserts (Simmons and Cirranello 2020). This remarkable diversity reflects their ecological adaptability and highlights their essential roles as pollinators, seed dispersers, and insect predators (Kunz et al. 2011).

However, bats are also recognized as reservoirs for a wide range of pathogens, including viruses, bacteria, and protozoan parasites (Brook and Dobson 2015). Among these, trypanosomes, a group of flagellate protozoans, are of particular interest due to their significant impact on public health and livestock productivity (Castillo-Castañeda et al. 2022; Austen and Barbosa 2021). The association between bats and trypanosomes has raised concerns about their role as potential reservoirs, contributing to parasite transmission dynamics (Dos Santos et al. 2018; Lima et al. 2013).

In addition to the well-known pathogens *Trypanosoma cruzi* and *T. evansi*, approximately 50 trypanosome MOTUs (molecular operational taxonomic unit; species and genotypes) have been reported in nearly one hundred bat species across the Americas, Africa, Asia, Europe, and Oceania (e.g., Lima et al. 2012; Aregawi et al. 2019; Dario et al. 2021), indicating a long co-evolutionary history and highlighting bats as important hosts (Lima et al. 2012, 2013; Ramírez et al. 2014; Riana et al. 2022). This diversity also underscores the need for ongoing surveillance and research to assess the potential of bat trypanosomes to emerge as zoonotic pathogens (Austen and Barbosa 2021; Juárez-Gabriel et al. 2024). Molecular techniques have been instrumental in uncovering new species (Hamilton et al. 2004) and elucidating relationships among known ones (Kostygov et al. 2021). These advancements have led to the recognition of bats as key reservoirs for diverse trypanosome species (Lima et al. 2012, 2013; Ramírez et al. 2014), some of which may have zoonotic relevance (Lima et al. 2013; Austen and Barbosa 2021). This makes it essential to study these parasites across diverse ecological settings and in relation to potential insect vectors (Riana et al. 2022).

The central part of the African continent remains among the most understudied regions of the world in terms of overall biodiversity and, in particular, potentially zoonotic pathogens. Countries like Uganda or the Central African Republic (CAR) exhibit diverse vegetation due to their unique ecological positions. Uganda features a mosaic of ecosystems, shaped by its location at the intersection of several African bioregions (Linder et al. 2012). Similarly, CAR’s vegetation includes tropical forests, savannas, and wetlands, reflecting substantial ecological heterogeneity, yet its fauna (including parasites) remains significantly underexplored.

In Uganda, which harbors nearly one hundred bat species (Babyesiza et al., under review), the study of bat-associated trypanosomes is particularly relevant given the country’s longstanding history with trypanosomiasis (Berrang-Ford et al. 2006; Welburn et al. 2016). Similarly, the Central African Republic (CAR), formerly known as Oubangi-Chari, also has a tragic past linked to human African trypanosomiasis. Alongside the devastating impacts of the trans-Saharan and trans-Atlantic slave trades, the disease likely contributed to large-scale depopulation, particularly in the eastern regions of the country (Franco et al. 2022). Despite continued challenges posed by political instability and limited infrastructure, trypanosomiasis remains a public health concern in parts of CAR even today.

Bats in CAR represent a diverse but poorly studied component of the country’s mammalian fauna (Schlitter et al. 1982). Preliminary fieldwork and acoustic monitoring suggest the presence of a rich assemblage of both insectivorous and frugivorous species, with a high probability of cryptic or yet undescribed taxa, particularly given the presence of closely related species in adjacent countries. Given their potential role as reservoirs for zoonotic pathogens such as Ebola and Marburg viruses, improving our understanding of bat diversity in CAR is essential for both conservation biology and public health preparedness.

This study contributes to the documentation of trypanosome diversity in bat populations from East and Central Africa, with a focus on host–parasite associations and novel host records. Our findings provide insights into parasite sharing, host specificity, and the evolutionary dynamics of trypanosomes within African bat communities.

## Materials and methods

### Study area

**Fig. 1:**
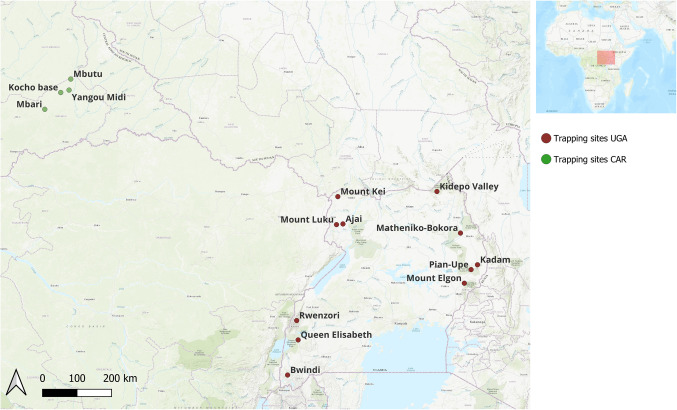
View of bat sampling sites in the Central African Republic (green circles) and Uganda (red circles)

The study was conducted in two geographically distinct regions (Fig. 1), Uganda and eastern CAR, with independent sampling periods. In Uganda (2021–2022), bats were sampled across diverse habitats, including grasslands, fallows, woodlands, and forests in Karamoja, West Nile, and the Albertine Rift. Key sites included Kidepo Valley NP; Mounts Moroto (Matheniko-Bokora), Kadam, and Elgon; West Nile forest reserves (Mounts Kei and Luku, Ajai); and tropical lowland forests (Mabira Forest, Queen Elizabeth NP) and montane (Rwenzori, Bwindi) forests. In the CAR (2024), sampling occurred in the Chinko Conservation Area across four localities: Kocho base, Yangou Midi, Mbari, and Mbutu. Mist nets were set over rivers, streams, and forest trails from 7 pm for 6 h, supplemented by roost-site targeting (caves, abandoned latrines, buildings). This comprehensive strategy aimed to maximize bat species diversity across ecological settings.

### DNA extraction, amplification, sequencing

DNA was extracted from tissue preserved in 96% ethanol and from dried blood spot samples following the manufacturer’s protocol. Host species were identified via DNA barcoding by amplifying and sequencing the mitochondrial cytochrome b (cyt-*b*) gene, following Bryja et al. (2014). Resulting sequences were compared to unpublished reference data held at the Institute of Vertebrate Biology, Czech Academy of Sciences, as well as to sequences from recent taxonomic and phylogenetic studies available in GenBank.

Trypanosome 18S rRNA genes were amplified using a trypanosomatid-specific nested PCR protocol (Seward et al. 2017), with ~ 10 ng of DNA per reaction. PCR products were directly sequenced, and raw sequences were processed and analyzed in Geneious v.2025.0.3 (https://www.geneious.com).

### Phylogenetic and host–parasite association analysis

Phylogenetic reconstruction was performed using pairwise deletion of positions containing gaps or missing data. The best-fitting substitution model was selected using ModelFinder under the Bayesian Information Criterion (BIC), as implemented in IQ-TREE. Phylogenetic analyses were carried out using maximum likelihood (ML) in PhyML with the GTR + G + I model and 1000 bootstrap replicates, and Bayesian inference (BI) in MrBayes, with 5 million generations and the covarion model.

A host–parasite matrix was generated to map trypanosome lineages across bat species, highlighting consistent and novel host associations.

## Results

A total of 164 bats representing 33 species (verified via cyt-*b* sequencing) were collected and screened for trypanosomes using nested PCR: 85 individuals (18 species) from Uganda and 79 (19 species) from CAR (Table 1; S1). Trypanosome prevalence was 36.5% in Uganda (31/85) and 13.9% in CAR (11/79). Phylogenetic analysis was performed on a multiple alignment of 64 mostly full-length 18S rRNA sequences. Newly obtained sequences (in total 42 representing 22 different genotypes) were approximately 2025 bp in length after primer trimming. They were complemented by the GenBank sequences that ranged from 492 to 2154 bp. The final alignment comprised 2252 nucleotides: 474 variable sites, 1778 conserved sites (79.0%), and an average pairwise identity of 91.4%.

**Table 1:** Trypanosome infection rates and genotypic diversity in bats from Uganda and the Central African Republic

Bat species	Total/Pos	Country	*Trypanosoma* species (genotype)
*Afronycteris nana*	4/2	CAR	*dionisii* (4), *vespertilionis* (3a)
	2	Uganda	
*Chaerephon leucogaster*	2	Uganda	
*Chaerephon ansorgei*	10	Uganda	
*Doryrhina cyclops*	2/1	CAR	*vespertilionis* (AFF)
*Eidolon helvum*	2	CAR	
*Epomophorus gambianus*	10	CAR	
*Epomops franqueti*	2	Uganda	
*Hipposideros caffer*	7/1	CAR	*livingstonei* (3b)
	3/3	Uganda	*livingstonei* (AFF)
*Hipposideros ruber*	1/1	CAR	*livingstonei* (3a)
	2	Uganda	
*Laephotis kirinyaga*	6/2	CAR	*dionisii* (3), *vespertilionis* (3b)
*Lissonycteris angolensis*	5/3	Uganda	‘flying-fox’ (1,3)
*Macronycteris gigas*	6	CAR	
*Micropteropus pusillus*	22	CAR	
*Mimetillus moloneyi*	1	CAR	
*Mops condylurus*	14/4	Uganda	*erneyi* (1)*, livingstonei* (1a,1b)
*Myonycteris torquata*	3	CAR	
*Myotis tricolor*	1	Uganda	
*Neoromicia somalicus*	2	Uganda	
*Nycteris macrotis*	1/1	Uganda	*livingstonei* (2)
*Nycteris thebaica*	2/1	Uganda	*livingstonei* (4)
*Nycticeinops crassulus*	2	CAR	
*Pipistrellus* cf.* nanulus*	1	CAR	
*Pipistrellus hesperidus*	5/4	Uganda	*dionisii* (1,2), *vespertilionis* (1,2)
*Pipistrellus* sp. 1	1	CAR	
*Pipistrellus* sp. 2	1	CAR	
*Rhinolophus eloquens*	3/3	Uganda	*livingstonei* (1b)
*Rhinolophus fumigatus*	2	CAR	
*Rhinolophus landeri*	8/4	Uganda	*livingstonei* (1a)
*Rhinolophus ruwenzorii*	4	Uganda	
*Rousettus aegyptiacus*	18/8	Uganda	*livingstonei* (1b), ‘flying-fox’ (1,2,4)
*Scotoecus hindei*	5/4	CAR	*vespertilionis* (4)
*Scotophilus dingani*	1	CAR	
*Taphozous mauritianus*	2	CAR	
	1	Uganda	

Our phylogenetic analysis of 18S rRNA gene sequences revealed seven trypanosome phylogroups, represented by 22 genotypes (Table 1 and Fig. 1): *Trypanosoma dionisii* (4 genotypes), *Trypanosoma erneyi* (1), *Trypanosoma livingstonei* (6), *Trypanosoma* aff. *livingstonei* (1; *Hipposideros caffer*), *Trypanosoma vespertilionis* (5), *Trypanosoma* aff. *vespertilionis* (1; *Doryrhina cyclops*), and a new clade, *Trypanosoma* sp. “flying-fox” (4), found exclusively in Pteropodidae. Except for five previously described genotypes—*T. livingstonei* 3a (PQ868320; *Hipposideros ruber*), *T. livingstonei* 2 (PQ868319; *Nycteris macrotis*), *T.* aff. *livingstonei* (PQ868323; *Hipposideros caffer*), *T. dionisii* 2 (PQ868305; *Pipistrellus hesperidus*), and *T. erneyi* (PQ868308; *Mops condylurus*)—all detected genotypes are novel.

**Fig. 2:**
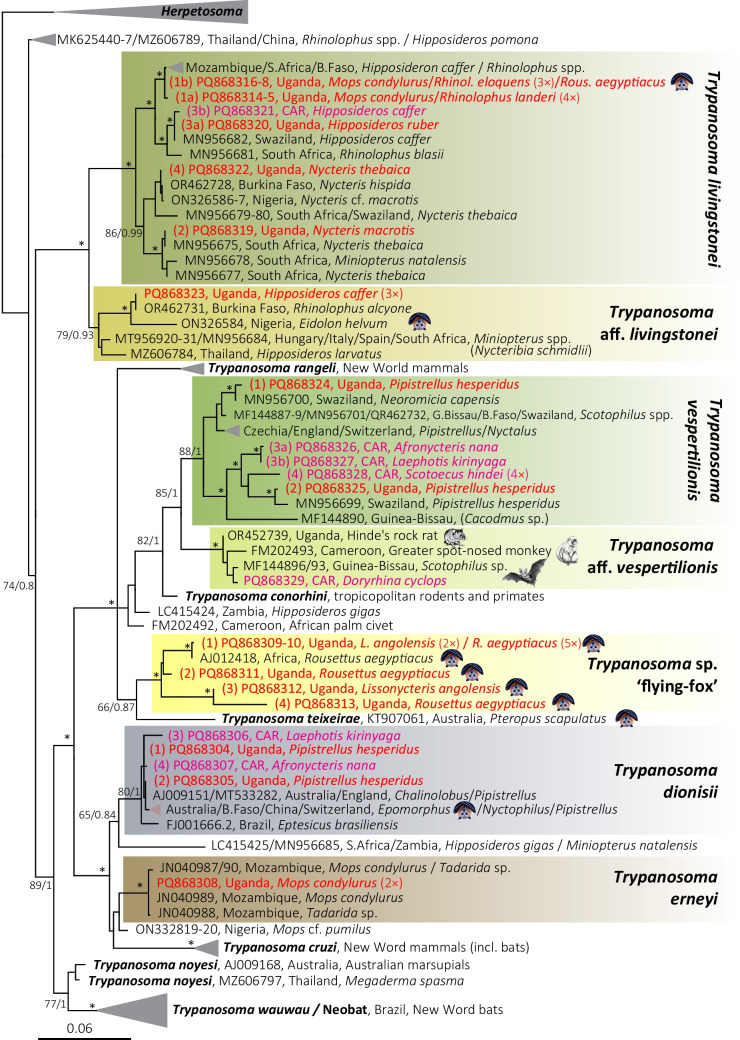
Maximum likelihood phylogeny of *Trypanosoma* spp. from bats based on 18S rRNA sequences. Host species and geographic origin are indicated; sequences from this study are color-coded (Uganda: red; CAR: purple). Fruit bats (Pteropodidae) are marked with an upside-down bat icon. Node support is shown as bootstrap values (ML) and posterior probabilities (BI); asterisks denote maximal support (ML > 90, BI > 0.95). The scale bar indicates genetic divergence

Six genotypes of *Trypanosoma livingstonei* were detected in multiple bat species, including *Hipposideros caffer*, *H. ruber*, *Mops condylurus*, *Nycteris macrotis*, *N. thebaica, Rhinolophus eloquens*, *R. landeri,* and *Rousettus aegyptiacus,* with the highest prevalence in *Rhinolophus* spp*.* Five genotypes of *Trypanosoma vespertilionis* were found in *Afronycteris nana*, *Laephotis kirinyaga*, *Scotoecus hindei*, and *Pipistrellus hesperidus*. Four genotypes of *Trypanosoma dionisii* were detected in *Afronycteris nana*, *Laephotis kirinyaga*, and *Pipistrellus hesperidus* (Figs. 2 and S1)*.*

A new clade, *Trypanosoma* sp. “flying-fox,” comprising four genotypes from fruit bats (Pteropodidae), was recorded exclusively in Ugandan bats, despite nearly half of all screened CAR bats being pteropodids. This clade, which is sister to *T. teixeirae* from *Pteropus scapulatus* (Australian), exhibits intra-clade divergence of ~ 0.01–0.015 (Fig. 2), indicating considerable genetic diversity.

Three trypanosome species were each represented by a single genotype: *T. erneyi* was detected in two *Mops condylurus*; an unnamed species, closely related to *T. livingstonei*, infected three *Hipposideros caffer,* and one *Doryrhina cyclops* hosted a trypanosome species (designated in the phylogenetic tree as *Trypanosoma* aff. *vespertilionis*) previously reported in various African mammals (Fig. 2 and Fig. S1).

## Discussion

Our study provides new insights into the diversity and phylogenetic relationships of bat trypanosomes, revealing several novel species, genotypes, and host associations. We identified 22 genotypes clustered into seven phylogroups (putative distinct species), underscoring the genetic diversity of bat trypanosomes. This finding is consistent with previous research reporting considerable trypanosome diversity in bats globally (Austen and Barbosa 2021; Dario et al. 2017; Lima et al. 2013). The three most widespread species, *T. dionisii* (subgenus *Schizotrypanum*), *T. livingstonei*, and *T. vespertilionis* (subgenus *Aneza*) (Hamilton et al. 2012; Austen and Barbosa 2021), were each represented by multiple, mostly newly identified genotypes. In contrast, only a single, previously described genotype of *T. erneyi* (*Schizotrypanum*) was found. *Trypanosoma dionisii* has a cosmopolitan distribution, with recent records in Asian and Australian bats (Austen and Barbosa 2021), while *T. erneyi* and *T. livingstonei* appear to be restricted to sub-Saharan Africa. The trypanosome *T. livingstonei* was relatively recently described in bats from Mozambique (Lima et al. 2013), and since then, various genotypes belonging to the *T. livingstonei* phylogroup have been reported from a range of African bat species (Tsague et al. 2024).

Although we detected these trypanosomes in several new hosts, this is not surprising, given the previously reported broad host specificity (Lima et al. 2013; Thiombiano et al. 2023; Riana et al. 2022; Tsague et al. 2024). In contrast, the detection of *T. erneyi* in only two individuals of *Mops condylurus* is noteworthy, as it corroborates previous findings and further supports its high host specificity to the genera *Mops* and *Tadarida* (subfamily Molossinae).

Based on the phylogenetic analysis, it is noteworthy that within both *T. livingstonei* and *T. vespertilionis*, the analyzed sequences segregate into two distinct phyloclades. However, this subdivision does not correlate with the geographic origin of the sequences, suggesting that either these trypanosome species maintain high genetic diversity due to large effective population sizes or that the observed subgroups represent distinct subspecies or even separate species. This pattern is particularly striking when contrasted with the relatively low genetic diversity observed in *T. dionisii*, whose sequences, despite originating from both the Old and New Worlds, form a remarkably homogeneous clade (Fig. 2).

However, the most intriguing sequences are those obtained from fruit bats (Pteropodidae), with the exception of a single instance of *T. livingstonei*, form a distinct phylogroup exclusive to fruit bats. This phylogroup exhibits high genetic diversity and is sister to the Australian fruit bat (*Pteropus scapulatus*) trypanosome *T. teixeirae*. Given its distinct phylogenetic position, this newly identified phylogroup is a strong candidate for recognition as a new species. Unexpectedly, trypanosomes were detected only in Ugandan fruit bats, despite the fact that nearly half of the screened bats from CAR were also fruit bats. This suggests that ecological or evolutionary factors may influence parasite distribution. A comparable pattern was observed by Cavazzana et al. (2010), who found geographically structured clades of *T. cruzi*, *T. cruzi marinkellei*, and *T. cf. dionisii* in bats across different Brazilian biomes, with strong host and biome associations, indicating that ecological context and geographic separation shape trypanosome diversity.

The host specificity of *Trypanosoma* is often driven more by ecological than taxonomic factors, with vector specificity and ecology playing a key role. With the exception of *T. cruzi marinkellei*, confirmed vectors of bat trypanosomes remain largely unknown. However, it is generally assumed that permanent ectoparasites (e.g., Nycteribiidae, Streblidae, Polyctenidae), nest-associated parasites (e.g., Cimicidae, Triatominae), and blood-feeding flying insects (micropredators such as sand flies, biting midges, and stable or horse flies) may be involved in transmission (Austen and Barbosa 2021). In bats, vector ecology and behavior may constrain host infection patterns more significantly than the parasite’s intrinsic host preference. For bat trypanosomes, ecological interactions such as roosting behavior and host co-occurrence may be as important—or even more so—than host taxonomy. Roost sharing and sociality enhance exposure to blood-feeding vectors, especially in dense colonies or confined roosts (e.g., toilet boxes), thereby facilitating parasite transmission. We propose that regional differences in *Trypanosoma* prevalence and diversity likely reflect differences in environmental conditions, including topography, biogeographical position, pollution levels, habitat fragmentation, and other ecological pressures.

While all known sequences of the four formally described species (and their corresponding phylogroups), along with one as-yet undescribed fruit bat trypanosome species, have so far been found exclusively in bats, the trypanosome identified in the cyclops leaf-nosed bat (*Doryrhina cyclops*) belongs to a particularly intriguing phylogroup. This clade includes sequences from the greater spot-nosed monkey (*Cercopithecus nictitans*) in Cameroon, Hinde’s rock rat (*Aethomys hindei*) in Uganda (Babyesiza et al. 2024), and a yellow bat (*Scotophilus* sp.) in Guinea-Bissau. These findings suggest that this species is ecologically well-adapted to tropical Africa, where it maintains a broad vertebrate host range and is capable of infecting multiple mammalian groups.

The apparent benignity of most bat trypanosomes to their hosts is of epidemiological relevance, as the absence of severe pathology enhances the reservoir competence of bats. An additional factor contributing to the role of bats in maintaining and disseminating *Trypanosoma* spp. across ecosystems is their long lifespan, which allows infections to persist for years.

## Conclusion

Our findings expand the current understanding of bat trypanosome diversity by revealing a rich assemblage of genotypes and previously unrecognized phylogenetic lineages. The detection of 22 genotypes across seven phylogroups, many of which represent novel host associations and potential new species, highlights the complexity and evolutionary depth of these parasites within chiropteran hosts. The consistent identification of known species such as *T. dionisii*, *T. livingstonei*, and *T. vespertilionis* alongside the limited but specific detection of *T. erneyi* supports a pattern of both widespread and host-restricted trypanosome lineages.

The phylogenetic structure, particularly the distinct subclades within *T. livingstonei* and *T. vespertilionis*, and the exclusive fruit bat-associated phylogroup related to *T. teixeirae*, emphasizes the need for taxonomic revision and further investigation into potential cryptic species. The striking absence of trypanosomes in Central African Republic fruit bats, contrasted with the rich diversity observed in Ugandan populations, suggests that ecological, geographical, or evolutionary forces may be shaping the distribution of these parasites.

Collectively, this study underscores the importance of continued surveillance and phylogenetic characterization of bat trypanosomes to unravel their biodiversity, host specificity, and biogeographic patterns.

## Supplementary information

Figure S1. Matrix showing the distribution of *Trypanosoma* species across bat species. Dark cells indicate the presence of a given *Trypanosoma* lineage in a particular host. Bat species are listed on the y-axis and *Trypanosoma* lineages on the x-axis, highlighting patterns of host–parasite associations and lineage specificity. Bat species are sorted phylogenetically, color-coded by family, and grouped according to the two major bat suborders: Yinpterochiroptera (marked in red) and Yangochiroptera (marked in green).

Below is the link to the electronic supplementary material.

Supplementary file (PNG 87.4 KB)

## Data Availability

The sequence data supporting the findings of this study have been deposited in GenBank under accession numbers PQ868304–PQ868329.
