# Supplementary figures and images for: Hidden diversity and host specificity of bat trypanosomes in East and Central Africa
Source: Parasitol Res. 2025 Sep 11;124(9):101. doi: 10.1007/s00436-025-08547-4 (PMC12425843; doi:10.1007/s00436-025-08547-4)

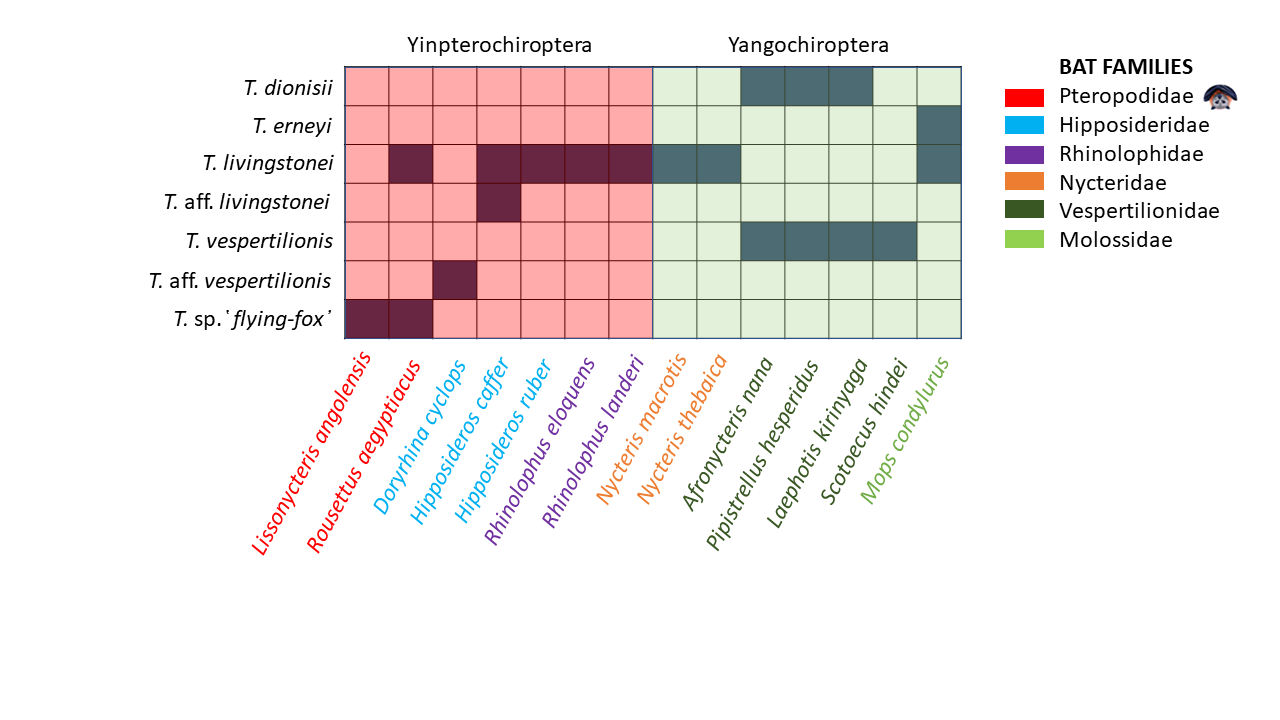

Supplement: Supplementary file 1 — Supplementary file (PNG 87.4 KB) [file 436_2025_8547_MOESM1_ESM.png]
